# (2,2′-Bipyridine-4,4′-dicarb­oxy­lic acid-κ^2^ *N*,*N*′)chlorido(2,2′:6′,2′′-terpyridyl-κ^3^ *N*,*N*′,*N*′′)ruthenium(II) perchlorate ethanol monosolvate monohydrate

**DOI:** 10.1107/S1600536811054195

**Published:** 2011-12-23

**Authors:** Anne Nielsen, Christine J. McKenzie, Andrew D. Bond

**Affiliations:** a University of Southern Denmark, Department of Physics and Chemistry, Campusvej 55, 5230 Odense, Denmark

## Abstract

In the title compound, [RuCl(C_15_H_11_N_3_)(C_12_H_8_N_2_O_4_)]ClO_4_·C_2_H_5_OH·H_2_O, the geometry of the ClN_5_ coordination set around the Ru^II^ atom is close to octa­hedral, but distorted on account of the limited bite angles of the polypyridyl ligands. The complexes are linked by O—H⋯O hydrogen bonds between the carboxyl groups and the crystal lattice water mol­ecules into chains along [110]. Face-to-face stacking inter­actions are formed between terpyridine ligands, with inter­planar separations of 3.66 (1) and 3.42 (1) Å, and between bipyridine-4,4′-dicarb­oxy­lic acid ligands, with inter­planar separations of 3.65 (1) and 3.72 (1) Å. Three O atoms of the perchlorate ion are each disordered equally over two positions. The hy­droxy group of the ethanol mol­ecule is also disordered over two sites with refined occupancies of 0.794 (9) and 0.206 (9).

## Related literature

For background literature concerning Ru^II^ complexes containing polypyridyl ligands, see: Kalyanasundaram (1982 ▶); Juris *et al.* (1988 ▶); Concepcion *et al.* (2008 ▶). For some other Ru^II^ complexes containing the 2,2′-bipyridine-4,4′-dicarb­oxy­lic acid-*N*,*N*′ ligand, see: Caspar *et al.* (2004 ▶); Eskelinen *et al.* (2000 ▶); Fujihara *et al.* (2004 ▶); Pearson *et al.* (2008 ▶); Philippopoulos *et al.* (2007 ▶). Synthesis details for the precursor RuCl_3_(terpy) are given in Takeuchi *et al.* (1984 ▶).
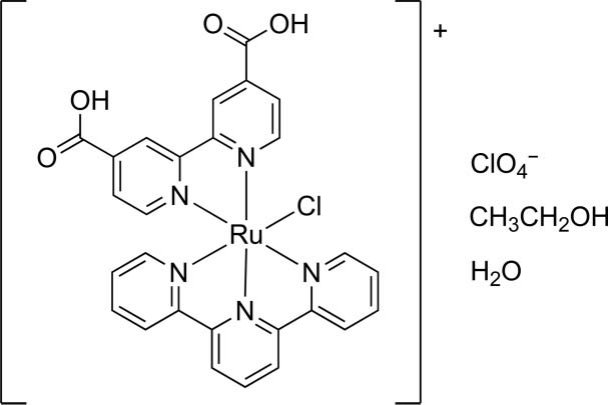


## Experimental

### 

#### Crystal data

[RuCl(C_15_H_11_N_3_)(C_12_H_8_N_2_O_4_)]ClO_4_·C_2_H_6_O·H_2_O
*M* *_r_* = 777.53
Triclinic, 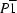

*a* = 8.7132 (5) Å
*b* = 11.9207 (7) Å
*c* = 15.9015 (8) Å
α = 90.913 (2)°
β = 104.110 (2)°
γ = 97.677 (2)°
*V* = 1585.44 (15) Å^3^
*Z* = 2
Mo *K*α radiation
μ = 0.73 mm^−1^
*T* = 180 K
0.20 × 0.12 × 0.10 mm

#### Data collection

Bruker–Nonius X8 APEXII CCD diffractometer
Absorption correction: multi-scan (*SADABS*; Sheldrick, 2003 ▶) *T*_min_ = 0.792, *T*_max_ = 0.931
22443 measured reflections
5961 independent reflections
4978 reflections with *I* > 2σ(*I*)
*R*_int_ = 0.031

#### Refinement

*R*[*F* ^2^ > 2σ(*F* ^2^)] = 0.040
*wR*(*F* ^2^) = 0.112
*S* = 1.08
5961 reflections
462 parameters
73 restraints
H-atom parameters constrained
Δρ_max_ = 0.91 e Å^−3^
Δρ_min_ = −0.74 e Å^−3^

### 

Data collection: *APEX2* (Bruker–Nonius, 2004) ▶; cell refinement: *SAINT* (Bruker, 2003 ▶); data reduction: *SAINT*; program(s) used to solve structure: *SHELXTL* (Sheldrick, 2008 ▶); program(s) used to refine structure: *SHELXTL*; molecular graphics: *SHELXTL*; software used to prepare material for publication: *SHELXTL*.

## Supplementary Material

Crystal structure: contains datablock(s) global, I. DOI: 10.1107/S1600536811054195/is5031sup1.cif

Structure factors: contains datablock(s) I. DOI: 10.1107/S1600536811054195/is5031Isup2.hkl

Additional supplementary materials:  crystallographic information; 3D view; checkCIF report

## Figures and Tables

**Table 1:** Hydrogen-bond geometry (Å, °)

*D*—H⋯*A*	*D*—H	H⋯*A*	*D*⋯*A*	*D*—H⋯*A*
O2—H2⋯O1*S*	0.85	1.75	2.601 (5)	179
O2—H2⋯O1*T*	0.85	1.93	2.545 (18)	129
O4—H4⋯O1*W*^i^	0.85	1.72	2.569 (4)	179
O1*W*—H1*W*⋯O1^ii^	0.85	1.87	2.720 (4)	179
O1*W*—H2*W*⋯O2*C*^iii^	0.85	1.95	2.795 (12)	178
O1*S*—H1*S*⋯O2*A*^iv^	0.85	2.14	2.986 (8)	180
O1*T*—H1*T*⋯O2*D*^v^	0.85	1.84	2.69 (3)	180

Symmetry codes: (i) 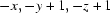; (ii) 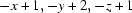; (iii) 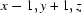; (iv) 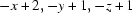; (v) 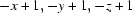.

**Fig. 1.**
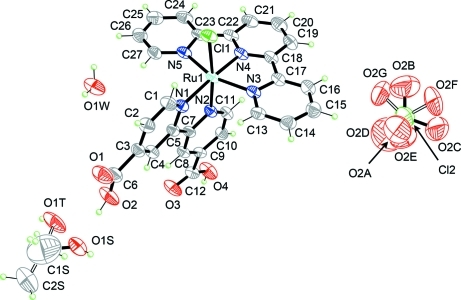
Molecular structure with displacement ellipsoids shown at 50% probability for non-H atoms.

**Fig. 2.**
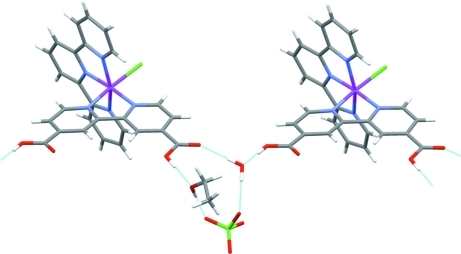
Hydrogen-bonded chains running along [110]. Only one orientaton is shown for the disordered ethanol molecule and perchlorate anion.

## supplementary crystallographic information

### Comment

Complexes of Ru^II^ with polypyridyl ligands are of interest on account of their photophysical and photochemical properties (Kalyanasundaram, 1982; Juris *et al.*, 1988). Recent work in the area of the catalysis of water oxidation has indicated the utility of single-site Ru complexes (Concepcion *et al.*, 2008). The next step towards realising artificial photosynthesis is to anchor these systems chemically to electrode materials. The title complex is intended to embody both required aspects: hydrolysis will result in replacement of the chloride ligand by hydroxide (the substrate of reaction), while the decorating carboxylate groups can be used as functionalities for linking the complex to surfaces.

### Experimental

The precursor RuCl_3_(terpy) was synthesized according to Takeuchi *et al.* (1984). RuCl_3_(terpy) (345 mg, 0.08 mmol), H_2_(bipy-dca)H (bipy-dca = 2,2'-bipyridine-4,4'-dicarboxylic acid, 191 mg, 0.8 mmol) and triethylamine (380 ml, 2.7 mmol) were mixed in ethanol (30 ml) and water (10 ml) and heated under reflux overnight. The reaction mixture was cooled to room temperature and a small amount of black precipitate was removed by filtration. The filtrate was adjusted to pH 2 with perchloric acid (70% aqueous solution) and orange crystals were deposited over 24 h.

### Refinement

H atoms bound to C atoms were positioned geometrically and allowed to ride during subsequent refinement, with C—H = 0.95 (aromatic), 0.98 (methyl) or 0.99 (methylene) Å, and with *U*_iso_(H) = 1.2 (aromatic, methylene) or 1.5 (methyl) *U*_eq_(C). H atoms bound to O atoms were positioned along the vector to the nearest hydrogen-bond acceptor with O—H = 0.85 Å, then allowed to ride with *U*_iso_(H) = 1.5 *U*_eq_(O). The perchlorate anion is modelled as disordered over two orientations with site occupancy 0.5. Atom O2A is common to both orientations. The Cl—O distances were restrained to a single refined value (1.395 Å), and O···O distances restrained to be 1.633 times that value, with standard uncertainty 0.01 Å for all restraints. All atoms were refined with anisotropic displacement parameters, with the disordered O atoms restrained to approximate isotropic behaviour. The ethanol molecule is modelled as disordered over two orientations, both in suitable positions to form hydrogen bonds to a neighbouring perchlorate anion. Atoms C1S/C1T in the two orientations were constrained to lie at the same coordinates with the same displacement parameters. All non-H atoms were refined anisotropically but restrained to approximate isotropic behaviour. The site occupancy factors for the two parts were constrained to sum to unity; the refined values are 0.794 (9):0.206 (9). The largest peak in the difference density lies close to the disordered ethanol molecule.

### Crystal data

**Table:**

[RuCl(C_15_H_11_N_3_)(C_12_H_8_N_2_O_4_)]ClO_4_·C_2_H_6_O·H_2_O	*Z* = 2
*M**_r_* = 777.53	*F*(000) = 788
Triclinic, *P*1	*D*_x_ = 1.629 Mg m^−^^3^
Hall symbol: -P 1	Mo *K*α radiation, λ = 0.71073 Å
*a* = 8.7132 (5) Å	Cell parameters from 8777 reflections
*b* = 11.9207 (7) Å	θ = 2.5–25.5°
*c* = 15.9015 (8) Å	µ = 0.73 mm^−^^1^
α = 90.913 (2)°	*T* = 180 K
β = 104.110 (2)°	Block, brown
γ = 97.677 (2)°	0.20 × 0.12 × 0.10 mm
*V* = 1585.44 (15) Å^3^	

### Data collection

**Table:**

Bruker–Nonius X8 APEXII CCD diffractometer	5961 independent reflections
Radiation source: fine-focus sealed tube	4978 reflections with *I* > 2σ(*I*)
graphite	*R*_int_ = 0.031
thin–slice ω and φ scans	θ_max_ = 25.7°, θ_min_ = 3.6°
Absorption correction: multi-scan (*SADABS*; Sheldrick, 2003)	*h* = −10→10
*T*_min_ = 0.792, *T*_max_ = 0.931	*k* = −14→14
22443 measured reflections	*l* = −19→17

### Refinement

**Table:**

Refinement on *F*^2^	Primary atom site location: structure-invariant direct methods
Least-squares matrix: full	Secondary atom site location: difference Fourier map
*R*[*F*^2^ > 2σ(*F*^2^)] = 0.040	Hydrogen site location: inferred from neighbouring sites
*wR*(*F*^2^) = 0.112	H-atom parameters constrained
*S* = 1.08	*w* = 1/[σ^2^(*F*_o_^2^) + (0.0608*P*)^2^ + 1.3719*P*] where *P* = (*F*_o_^2^ + 2*F*_c_^2^)/3
5961 reflections	(Δ/σ)_max_ = 0.001
462 parameters	Δρ_max_ = 0.91 e Å^−^^3^
73 restraints	Δρ_min_ = −0.74 e Å^−^^3^

### Special details

**Table:**

Geometry. All e.s.d.'s (except the e.s.d. in the dihedral angle between two l.s. planes) are estimated using the full covariance matrix. The cell e.s.d.'s are taken into account individually in the estimation of e.s.d.'s in distances, angles and torsion angles; correlations between e.s.d.'s in cell parameters are only used when they are defined by crystal symmetry. An approximate (isotropic) treatment of cell e.s.d.'s is used for estimating e.s.d.'s involving l.s. planes.
Refinement. Refinement of *F*^2^ against ALL reflections. The weighted *R*-factor *wR* and goodness of fit *S* are based on *F*^2^, conventional *R*-factors *R* are based on *F*, with *F* set to zero for negative *F*^2^. The threshold expression of *F*^2^ > σ(*F*^2^) is used only for calculating *R*-factors(gt) *etc*. and is not relevant to the choice of reflections for refinement. *R*-factors based on *F*^2^ are statistically about twice as large as those based on *F*, and *R*- factors based on ALL data will be even larger.

### Fractional atomic coordinates and isotropic or equivalent isotropic displacement parameters (Å^2^)

**Table:**

	*x*	*y*	*z*	*U*_iso_*/*U*_eq_	Occ. (<1)
Ru1	0.30449 (3)	0.52232 (3)	0.229857 (17)	0.03172 (12)	
Cl1	0.38277 (11)	0.64610 (8)	0.12517 (6)	0.0394 (2)	
O1	0.6470 (4)	0.9485 (3)	0.5612 (2)	0.0648 (9)	
O2	0.5089 (4)	0.8261 (3)	0.6277 (2)	0.0655 (9)	
H2	0.5578	0.8627	0.6751	0.098*	
O3	0.0613 (4)	0.3562 (3)	0.59862 (19)	0.0588 (9)	
O4	−0.0105 (4)	0.1927 (3)	0.5215 (2)	0.0648 (9)	
H4	−0.0493	0.1692	0.5632	0.097*	
N1	0.3926 (3)	0.6406 (3)	0.33304 (19)	0.0334 (7)	
N2	0.2417 (3)	0.4362 (3)	0.32745 (19)	0.0329 (7)	
N3	0.5009 (4)	0.4411 (3)	0.23588 (19)	0.0348 (7)	
N4	0.2151 (4)	0.4007 (3)	0.14012 (18)	0.0345 (7)	
N5	0.0728 (4)	0.5593 (3)	0.18561 (19)	0.0361 (7)	
C1	0.4864 (5)	0.7410 (3)	0.3332 (2)	0.0404 (9)	
H1A	0.5156	0.7623	0.2813	0.048*	
C2	0.5406 (5)	0.8132 (3)	0.4058 (3)	0.0409 (9)	
H2A	0.6078	0.8823	0.4040	0.049*	
C3	0.4968 (5)	0.7846 (3)	0.4814 (2)	0.0374 (9)	
C4	0.4015 (4)	0.6820 (3)	0.4819 (2)	0.0365 (8)	
H4A	0.3699	0.6603	0.5331	0.044*	
C5	0.3525 (4)	0.6115 (3)	0.4079 (2)	0.0316 (8)	
C6	0.5576 (5)	0.8626 (4)	0.5610 (3)	0.0463 (10)	
C7	0.2617 (4)	0.4974 (3)	0.4037 (2)	0.0317 (8)	
C8	0.2015 (4)	0.4523 (3)	0.4706 (2)	0.0329 (8)	
H8A	0.2124	0.4975	0.5220	0.039*	
C9	0.1257 (4)	0.3418 (3)	0.4633 (2)	0.0347 (8)	
C10	0.1133 (4)	0.2776 (3)	0.3876 (2)	0.0370 (8)	
H10A	0.0654	0.2006	0.3814	0.044*	
C11	0.1712 (4)	0.3272 (3)	0.3219 (2)	0.0376 (9)	
H11A	0.1613	0.2827	0.2702	0.045*	
C12	0.0563 (5)	0.2980 (3)	0.5357 (2)	0.0395 (9)	
C13	0.6499 (4)	0.4691 (3)	0.2879 (2)	0.0384 (9)	
H13A	0.6713	0.5352	0.3254	0.046*	
C14	0.7705 (5)	0.4068 (4)	0.2889 (3)	0.0450 (10)	
H14A	0.8734	0.4290	0.3265	0.054*	
C15	0.7406 (5)	0.3105 (4)	0.2340 (3)	0.0480 (10)	
H15A	0.8219	0.2646	0.2345	0.058*	
C16	0.5917 (5)	0.2826 (4)	0.1791 (3)	0.0448 (10)	
H16A	0.5704	0.2184	0.1397	0.054*	
C17	0.4732 (5)	0.3474 (3)	0.1807 (2)	0.0374 (8)	
C18	0.3086 (5)	0.3238 (3)	0.1254 (2)	0.0388 (9)	
C19	0.2449 (5)	0.2350 (4)	0.0640 (3)	0.0482 (10)	
H19A	0.3095	0.1813	0.0526	0.058*	
C20	0.0859 (5)	0.2262 (4)	0.0198 (3)	0.0540 (11)	
H20A	0.0405	0.1650	−0.0216	0.065*	
C21	−0.0074 (5)	0.3047 (4)	0.0351 (3)	0.0467 (10)	
H21A	−0.1166	0.2982	0.0044	0.056*	
C22	0.0595 (4)	0.3930 (3)	0.0957 (2)	0.0380 (9)	
C23	−0.0195 (4)	0.4848 (3)	0.1210 (2)	0.0370 (9)	
C24	−0.1768 (5)	0.4973 (4)	0.0825 (3)	0.0464 (10)	
H24A	−0.2396	0.4451	0.0375	0.056*	
C25	−0.2408 (5)	0.5863 (4)	0.1104 (3)	0.0516 (11)	
H25A	−0.3483	0.5962	0.0846	0.062*	
C26	−0.1489 (5)	0.6602 (4)	0.1753 (3)	0.0495 (11)	
H26A	−0.1924	0.7214	0.1953	0.059*	
C27	0.0078 (5)	0.6455 (3)	0.2118 (2)	0.0402 (9)	
H27A	0.0713	0.6976	0.2567	0.048*	
Cl2	0.8935 (2)	0.01878 (13)	0.17808 (11)	0.0846 (4)	
O2A	1.0065 (6)	0.1063 (4)	0.2176 (3)	0.1302 (19)	
O2B	0.8067 (15)	0.0204 (11)	0.0971 (6)	0.166 (5)	0.50
O2C	0.9907 (13)	−0.0746 (7)	0.1832 (8)	0.135 (4)	0.50
O2D	0.8001 (17)	−0.0137 (14)	0.2390 (9)	0.233 (12)	0.50
O2E	0.8421 (13)	−0.0554 (9)	0.2330 (7)	0.116 (4)	0.50
O2F	0.9463 (14)	−0.0316 (9)	0.1130 (7)	0.143 (4)	0.50
O2G	0.7602 (10)	0.0775 (8)	0.1339 (7)	0.120 (4)	0.50
O1W	0.1323 (4)	0.8776 (3)	0.3538 (2)	0.0649 (9)	
H1W	0.2019	0.9317	0.3804	0.097*	
H2W	0.0882	0.8906	0.3017	0.097*	
O1S	0.6600 (6)	0.9364 (4)	0.7731 (3)	0.0729 (18)	0.794 (9)
H1S	0.7550	0.9243	0.7757	0.109*	0.794 (9)
C1S	0.6167 (16)	1.0330 (11)	0.8050 (8)	0.181 (5)	0.794 (9)
H2S	0.5003	1.0299	0.7802	0.217*	0.794 (9)
H3S	0.6694	1.0981	0.7796	0.217*	0.794 (9)
O1T	0.485 (3)	0.9761 (15)	0.7379 (11)	0.087 (9)	0.206 (9)
H1T	0.3954	0.9877	0.7453	0.131*	0.206 (9)
C1T	0.6167 (16)	1.0330 (11)	0.8050 (8)	0.181 (5)	0.206 (9)
H2T	0.6421	1.1071	0.7805	0.217*	0.206 (9)
H3T	0.7059	0.9909	0.8019	0.217*	0.206 (9)
C2S	0.6458 (13)	1.0616 (8)	0.8968 (6)	0.156 (4)	
H4S	0.6063	1.1332	0.9049	0.233*	
H5S	0.7609	1.0696	0.9236	0.233*	
H6S	0.5900	1.0013	0.9241	0.233*	

### Atomic displacement parameters (Å^2^)

**Table:**

	*U*^11^	*U*^22^	*U*^33^	*U*^12^	*U*^13^	*U*^23^
Ru1	0.02898 (18)	0.0418 (2)	0.02243 (17)	−0.00185 (12)	0.00673 (11)	−0.00504 (12)
Cl1	0.0347 (5)	0.0531 (6)	0.0295 (5)	−0.0012 (4)	0.0101 (4)	0.0001 (4)
O1	0.091 (3)	0.0474 (18)	0.0447 (18)	−0.0189 (18)	0.0104 (17)	−0.0117 (14)
O2	0.097 (3)	0.0547 (19)	0.0401 (18)	−0.0176 (18)	0.0235 (17)	−0.0175 (14)
O3	0.078 (2)	0.0578 (19)	0.0398 (17)	−0.0216 (16)	0.0298 (16)	−0.0117 (14)
O4	0.093 (3)	0.0508 (19)	0.0516 (19)	−0.0214 (17)	0.0374 (18)	−0.0060 (14)
N1	0.0284 (15)	0.0400 (17)	0.0299 (16)	0.0002 (13)	0.0063 (12)	−0.0012 (13)
N2	0.0285 (16)	0.0404 (17)	0.0283 (15)	0.0014 (13)	0.0067 (12)	−0.0052 (13)
N3	0.0321 (16)	0.0452 (18)	0.0262 (15)	0.0002 (14)	0.0086 (13)	−0.0022 (13)
N4	0.0328 (16)	0.0453 (18)	0.0241 (15)	−0.0017 (14)	0.0089 (12)	−0.0052 (13)
N5	0.0349 (17)	0.0479 (19)	0.0252 (15)	−0.0008 (14)	0.0106 (13)	0.0001 (13)
C1	0.041 (2)	0.044 (2)	0.033 (2)	−0.0052 (18)	0.0091 (17)	−0.0013 (17)
C2	0.039 (2)	0.038 (2)	0.040 (2)	−0.0062 (17)	0.0068 (17)	−0.0034 (17)
C3	0.040 (2)	0.037 (2)	0.033 (2)	0.0024 (17)	0.0058 (16)	−0.0048 (16)
C4	0.041 (2)	0.039 (2)	0.0299 (19)	0.0023 (17)	0.0100 (16)	−0.0026 (15)
C5	0.0281 (18)	0.0376 (19)	0.0283 (18)	0.0016 (15)	0.0073 (14)	−0.0015 (15)
C6	0.058 (3)	0.041 (2)	0.036 (2)	0.002 (2)	0.0069 (19)	−0.0059 (17)
C7	0.0280 (18)	0.040 (2)	0.0242 (17)	0.0028 (15)	0.0021 (14)	−0.0053 (15)
C8	0.0331 (19)	0.039 (2)	0.0242 (17)	0.0001 (16)	0.0053 (14)	−0.0048 (14)
C9	0.0340 (19)	0.043 (2)	0.0250 (18)	0.0026 (16)	0.0052 (15)	0.0005 (15)
C10	0.039 (2)	0.034 (2)	0.036 (2)	−0.0003 (16)	0.0081 (16)	−0.0031 (16)
C11	0.039 (2)	0.041 (2)	0.033 (2)	0.0010 (17)	0.0109 (16)	−0.0075 (16)
C12	0.039 (2)	0.045 (2)	0.032 (2)	−0.0023 (17)	0.0079 (16)	0.0006 (17)
C13	0.037 (2)	0.047 (2)	0.0283 (19)	0.0020 (17)	0.0062 (16)	−0.0014 (16)
C14	0.034 (2)	0.058 (3)	0.040 (2)	0.0058 (19)	0.0040 (17)	0.0020 (19)
C15	0.042 (2)	0.055 (3)	0.049 (2)	0.010 (2)	0.0122 (19)	0.000 (2)
C16	0.045 (2)	0.049 (2)	0.040 (2)	0.0041 (19)	0.0134 (18)	−0.0054 (18)
C17	0.040 (2)	0.042 (2)	0.0299 (19)	0.0008 (17)	0.0109 (16)	−0.0029 (16)
C18	0.039 (2)	0.047 (2)	0.0296 (19)	0.0006 (17)	0.0102 (16)	−0.0048 (16)
C19	0.053 (3)	0.050 (2)	0.040 (2)	0.002 (2)	0.012 (2)	−0.0126 (19)
C20	0.054 (3)	0.058 (3)	0.041 (2)	−0.005 (2)	0.002 (2)	−0.016 (2)
C21	0.042 (2)	0.057 (3)	0.033 (2)	−0.007 (2)	0.0036 (17)	−0.0053 (18)
C22	0.033 (2)	0.052 (2)	0.0259 (19)	−0.0044 (17)	0.0073 (15)	0.0001 (16)
C23	0.033 (2)	0.052 (2)	0.0246 (18)	−0.0035 (17)	0.0100 (15)	−0.0006 (16)
C24	0.034 (2)	0.072 (3)	0.032 (2)	−0.002 (2)	0.0100 (17)	0.0024 (19)
C25	0.034 (2)	0.084 (3)	0.041 (2)	0.013 (2)	0.0152 (19)	0.009 (2)
C26	0.044 (2)	0.067 (3)	0.042 (2)	0.014 (2)	0.018 (2)	0.002 (2)
C27	0.040 (2)	0.052 (2)	0.032 (2)	0.0059 (18)	0.0151 (17)	−0.0007 (17)
Cl2	0.1002 (12)	0.0709 (9)	0.0767 (10)	0.0131 (8)	0.0115 (9)	−0.0187 (8)
O2A	0.132 (4)	0.099 (3)	0.133 (4)	−0.013 (3)	0.002 (3)	−0.039 (3)
O2B	0.162 (9)	0.191 (10)	0.115 (8)	0.046 (8)	−0.030 (7)	−0.002 (7)
O2C	0.172 (8)	0.108 (6)	0.114 (7)	0.063 (6)	−0.008 (6)	−0.021 (6)
O2D	0.238 (15)	0.264 (15)	0.222 (14)	0.041 (10)	0.102 (10)	0.045 (10)
O2E	0.107 (7)	0.117 (7)	0.123 (7)	0.006 (6)	0.031 (6)	0.044 (6)
O2F	0.192 (9)	0.122 (7)	0.130 (8)	0.062 (7)	0.049 (7)	−0.053 (6)
O2G	0.112 (7)	0.122 (7)	0.129 (7)	0.068 (6)	0.012 (6)	0.000 (6)
O1W	0.084 (2)	0.060 (2)	0.0427 (17)	−0.0258 (17)	0.0212 (16)	−0.0058 (15)
O1S	0.090 (4)	0.064 (3)	0.055 (3)	0.007 (2)	0.002 (2)	−0.023 (2)
C1S	0.207 (9)	0.176 (8)	0.162 (8)	0.027 (7)	0.049 (7)	0.059 (7)
O1T	0.13 (2)	0.062 (12)	0.055 (11)	−0.032 (11)	0.017 (11)	−0.030 (9)
C1T	0.207 (9)	0.176 (8)	0.162 (8)	0.027 (7)	0.049 (7)	0.059 (7)
C2S	0.207 (8)	0.136 (6)	0.107 (6)	0.059 (6)	−0.008 (5)	−0.060 (5)

### Geometric parameters (Å, °)

**Table:**

Ru1—N1	2.072 (3)	C14—C15	1.387 (6)
Ru1—N2	2.019 (3)	C14—H14A	0.950
Ru1—N3	2.060 (3)	C15—C16	1.371 (6)
Ru1—N4	1.959 (3)	C15—H15A	0.950
Ru1—N5	2.079 (3)	C16—C17	1.374 (6)
Ru1—Cl1	2.4035 (9)	C16—H16A	0.950
O1—C6	1.201 (5)	C17—C18	1.476 (5)
O2—C6	1.297 (5)	C18—C19	1.388 (5)
O2—H2	0.850	C19—C20	1.381 (6)
O3—C12	1.197 (5)	C19—H19A	0.950
O4—C12	1.303 (5)	C20—C21	1.374 (6)
O4—H4	0.850	C20—H20A	0.950
N1—C1	1.356 (5)	C21—C22	1.382 (6)
N1—C5	1.357 (4)	C21—H21A	0.950
N2—C11	1.353 (5)	C22—C23	1.467 (6)
N2—C7	1.367 (4)	C23—C24	1.386 (5)
N3—C13	1.354 (5)	C24—C25	1.378 (6)
N3—C17	1.367 (5)	C24—H24A	0.950
N4—C22	1.357 (5)	C25—C26	1.364 (6)
N4—C18	1.358 (5)	C25—H25A	0.950
N5—C27	1.343 (5)	C26—C27	1.384 (6)
N5—C23	1.368 (5)	C26—H26A	0.950
C1—C2	1.374 (5)	C27—H27A	0.950
C1—H1A	0.950	Cl2—O2A	1.362 (4)
C2—C3	1.382 (5)	Cl2—O2B	1.327 (7)
C2—H2A	0.950	Cl2—O2C	1.478 (7)
C3—C4	1.384 (5)	Cl2—O2D	1.436 (8)
C3—C6	1.501 (5)	Cl2—O2E	1.362 (7)
C4—C5	1.379 (5)	Cl2—O2F	1.389 (7)
C4—H4A	0.950	Cl2—O2G	1.472 (6)
C5—C7	1.471 (5)	O1W—H1W	0.850
C7—C8	1.383 (5)	O1W—H2W	0.850
C8—C9	1.381 (5)	O1S—C1S	1.384 (14)
C8—H8A	0.950	O1S—H1S	0.850
C9—C10	1.389 (5)	C1S—C2S	1.447 (13)
C9—C12	1.497 (5)	C1S—H2S	0.990
C10—C11	1.376 (5)	C1S—H3S	0.990
C10—H10A	0.950	O1T—H1T	0.850
C11—H11A	0.950	C2S—H4S	0.980
C13—C14	1.363 (5)	C2S—H5S	0.980
C13—H13A	0.950	C2S—H6S	0.980
			
N4—Ru1—N2	95.74 (12)	C14—C13—H13A	118.5
N4—Ru1—N3	79.16 (12)	C13—C14—C15	119.0 (4)
N2—Ru1—N3	93.30 (12)	C13—C14—H14A	120.5
N4—Ru1—N1	174.59 (12)	C15—C14—H14A	120.5
N2—Ru1—N1	78.86 (12)	C16—C15—C14	118.9 (4)
N3—Ru1—N1	100.75 (11)	C16—C15—H15A	120.6
N4—Ru1—N5	79.40 (12)	C14—C15—H15A	120.6
N2—Ru1—N5	90.51 (11)	C15—C16—C17	120.2 (4)
N3—Ru1—N5	158.49 (12)	C15—C16—H16A	119.9
N1—Ru1—N5	100.76 (12)	C17—C16—H16A	119.9
N4—Ru1—Cl1	91.15 (9)	N3—C17—C16	121.2 (4)
N2—Ru1—Cl1	172.75 (9)	N3—C17—C18	114.3 (3)
N3—Ru1—Cl1	90.22 (9)	C16—C17—C18	124.5 (4)
N1—Ru1—Cl1	94.26 (9)	N4—C18—C19	119.8 (4)
N5—Ru1—Cl1	88.52 (8)	N4—C18—C17	112.6 (3)
C6—O2—H2	113.7	C19—C18—C17	127.6 (4)
C12—O4—H4	111.5	C20—C19—C18	118.8 (4)
C1—N1—C5	117.9 (3)	C20—C19—H19A	120.6
C1—N1—Ru1	126.9 (2)	C18—C19—H19A	120.6
C5—N1—Ru1	115.2 (2)	C21—C20—C19	120.8 (4)
C11—N2—C7	117.8 (3)	C21—C20—H20A	119.6
C11—N2—Ru1	126.0 (2)	C19—C20—H20A	119.6
C7—N2—Ru1	116.1 (2)	C20—C21—C22	119.2 (4)
C13—N3—C17	117.8 (3)	C20—C21—H21A	120.4
C13—N3—Ru1	127.7 (3)	C22—C21—H21A	120.4
C17—N3—Ru1	114.5 (2)	N4—C22—C21	120.0 (4)
C22—N4—C18	121.4 (3)	N4—C22—C23	112.7 (3)
C22—N4—Ru1	119.3 (3)	C21—C22—C23	127.4 (4)
C18—N4—Ru1	119.3 (2)	N5—C23—C24	121.2 (4)
C27—N5—C23	118.6 (3)	N5—C23—C22	115.5 (3)
C27—N5—Ru1	128.3 (3)	C24—C23—C22	123.3 (4)
C23—N5—Ru1	113.1 (2)	C25—C24—C23	119.2 (4)
N1—C1—C2	122.4 (4)	C25—C24—H24A	120.4
N1—C1—H1A	118.8	C23—C24—H24A	120.4
C2—C1—H1A	118.8	C26—C25—C24	119.6 (4)
C1—C2—C3	119.6 (4)	C26—C25—H25A	120.2
C1—C2—H2A	120.2	C24—C25—H25A	120.2
C3—C2—H2A	120.2	C25—C26—C27	119.7 (4)
C2—C3—C4	118.4 (3)	C25—C26—H26A	120.2
C2—C3—C6	119.6 (4)	C27—C26—H26A	120.2
C4—C3—C6	121.9 (3)	N5—C27—C26	121.8 (4)
C5—C4—C3	119.8 (3)	N5—C27—H27A	119.1
C5—C4—H4A	120.1	C26—C27—H27A	119.1
C3—C4—H4A	120.1	O2B—Cl2—O2E	119.5 (8)
N1—C5—C4	121.8 (3)	O2B—Cl2—O2A	123.0 (6)
N1—C5—C7	114.2 (3)	O2E—Cl2—O2A	114.9 (5)
C4—C5—C7	123.9 (3)	O2E—Cl2—O2F	114.3 (6)
O1—C6—O2	124.8 (4)	O2A—Cl2—O2F	108.7 (5)
O1—C6—C3	121.4 (4)	O2B—Cl2—O2D	113.6 (7)
O2—C6—C3	113.8 (4)	O2A—Cl2—O2D	106.4 (6)
N2—C7—C8	121.2 (3)	O2E—Cl2—O2G	109.1 (6)
N2—C7—C5	114.6 (3)	O2A—Cl2—O2G	102.5 (4)
C8—C7—C5	124.2 (3)	O2F—Cl2—O2G	106.3 (6)
C9—C8—C7	120.5 (3)	O2B—Cl2—O2C	108.5 (6)
C9—C8—H8A	119.8	O2A—Cl2—O2C	101.1 (5)
C7—C8—H8A	119.8	O2D—Cl2—O2C	101.5 (6)
C8—C9—C10	118.3 (3)	H1W—O1W—H2W	113.9
C8—C9—C12	118.7 (3)	C1S—O1S—H1S	125.9
C10—C9—C12	123.0 (3)	O1S—C1S—C2S	122.9 (9)
C11—C10—C9	119.1 (3)	O1S—C1S—H2S	106.6
C11—C10—H10A	120.4	C2S—C1S—H2S	106.6
C9—C10—H10A	120.4	O1S—C1S—H3S	106.6
N2—C11—C10	123.0 (3)	C2S—C1S—H3S	106.6
N2—C11—H11A	118.5	H2S—C1S—H3S	106.6
C10—C11—H11A	118.5	C1S—C2S—H4S	109.5
O3—C12—O4	125.1 (4)	C1S—C2S—H5S	109.5
O3—C12—C9	122.1 (4)	H4S—C2S—H5S	109.5
O4—C12—C9	112.8 (3)	C1S—C2S—H6S	109.5
N3—C13—C14	122.9 (4)	H4S—C2S—H6S	109.5
N3—C13—H13A	118.5	H5S—C2S—H6S	109.5
			
N4—Ru1—N1—C1	169.1 (11)	C4—C3—C6—O2	−1.8 (6)
N2—Ru1—N1—C1	171.8 (3)	C11—N2—C7—C8	−4.7 (5)
N3—Ru1—N1—C1	80.5 (3)	Ru1—N2—C7—C8	170.4 (3)
N5—Ru1—N1—C1	−99.8 (3)	C11—N2—C7—C5	174.5 (3)
Cl1—Ru1—N1—C1	−10.5 (3)	Ru1—N2—C7—C5	−10.4 (4)
N4—Ru1—N1—C5	−9.8 (14)	N1—C5—C7—N2	4.2 (4)
N2—Ru1—N1—C5	−7.1 (2)	C4—C5—C7—N2	−172.5 (3)
N3—Ru1—N1—C5	−98.4 (3)	N1—C5—C7—C8	−176.6 (3)
N5—Ru1—N1—C5	81.3 (3)	C4—C5—C7—C8	6.7 (6)
Cl1—Ru1—N1—C5	170.6 (2)	N2—C7—C8—C9	3.0 (5)
N4—Ru1—N2—C11	3.9 (3)	C5—C7—C8—C9	−176.2 (3)
N3—Ru1—N2—C11	−75.5 (3)	C7—C8—C9—C10	0.7 (5)
N1—Ru1—N2—C11	−175.8 (3)	C7—C8—C9—C12	−177.5 (3)
N5—Ru1—N2—C11	83.3 (3)	C8—C9—C10—C11	−2.4 (5)
Cl1—Ru1—N2—C11	165.6 (5)	C12—C9—C10—C11	175.7 (3)
N4—Ru1—N2—C7	−170.7 (3)	C7—N2—C11—C10	3.0 (5)
N3—Ru1—N2—C7	109.8 (3)	Ru1—N2—C11—C10	−171.6 (3)
N1—Ru1—N2—C7	9.5 (2)	C9—C10—C11—N2	0.6 (6)
N5—Ru1—N2—C7	−91.3 (3)	C8—C9—C12—O3	1.8 (6)
Cl1—Ru1—N2—C7	−9.1 (8)	C10—C9—C12—O3	−176.3 (4)
N4—Ru1—N3—C13	178.9 (3)	C8—C9—C12—O4	−179.4 (4)
N2—Ru1—N3—C13	−85.9 (3)	C10—C9—C12—O4	2.6 (5)
N1—Ru1—N3—C13	−6.6 (3)	C17—N3—C13—C14	−1.7 (6)
N5—Ru1—N3—C13	174.3 (3)	Ru1—N3—C13—C14	177.5 (3)
Cl1—Ru1—N3—C13	87.7 (3)	N3—C13—C14—C15	0.3 (6)
N4—Ru1—N3—C17	−1.9 (2)	C13—C14—C15—C16	1.6 (6)
N2—Ru1—N3—C17	93.3 (3)	C14—C15—C16—C17	−2.1 (6)
N1—Ru1—N3—C17	172.6 (2)	C13—N3—C17—C16	1.1 (5)
N5—Ru1—N3—C17	−6.5 (5)	Ru1—N3—C17—C16	−178.2 (3)
Cl1—Ru1—N3—C17	−93.0 (2)	C13—N3—C17—C18	−179.1 (3)
N2—Ru1—N4—C22	87.8 (3)	Ru1—N3—C17—C18	1.6 (4)
N3—Ru1—N4—C22	−179.9 (3)	C15—C16—C17—N3	0.7 (6)
N1—Ru1—N4—C22	90.5 (13)	C15—C16—C17—C18	−179.0 (4)
N5—Ru1—N4—C22	−1.6 (3)	C22—N4—C18—C19	−0.3 (6)
Cl1—Ru1—N4—C22	−89.9 (3)	Ru1—N4—C18—C19	177.8 (3)
N2—Ru1—N4—C18	−90.3 (3)	C22—N4—C18—C17	−179.8 (3)
N3—Ru1—N4—C18	2.0 (3)	Ru1—N4—C18—C17	−1.7 (4)
N1—Ru1—N4—C18	−87.6 (13)	N3—C17—C18—N4	0.0 (5)
N5—Ru1—N4—C18	−179.7 (3)	C16—C17—C18—N4	179.7 (4)
Cl1—Ru1—N4—C18	92.0 (3)	N3—C17—C18—C19	−179.5 (4)
N4—Ru1—N5—C27	−178.7 (3)	C16—C17—C18—C19	0.3 (7)
N2—Ru1—N5—C27	85.5 (3)	N4—C18—C19—C20	−0.8 (6)
N3—Ru1—N5—C27	−174.1 (3)	C17—C18—C19—C20	178.6 (4)
N1—Ru1—N5—C27	6.8 (3)	C18—C19—C20—C21	1.1 (7)
Cl1—Ru1—N5—C27	−87.3 (3)	C19—C20—C21—C22	−0.2 (7)
N4—Ru1—N5—C23	0.4 (2)	C18—N4—C22—C21	1.1 (5)
N2—Ru1—N5—C23	−95.3 (2)	Ru1—N4—C22—C21	−177.0 (3)
N3—Ru1—N5—C23	5.0 (5)	C18—N4—C22—C23	−179.5 (3)
N1—Ru1—N5—C23	−174.1 (2)	Ru1—N4—C22—C23	2.4 (4)
Cl1—Ru1—N5—C23	91.8 (2)	C20—C21—C22—N4	−0.8 (6)
C5—N1—C1—C2	−0.2 (6)	C20—C21—C22—C23	179.9 (4)
Ru1—N1—C1—C2	−179.1 (3)	C27—N5—C23—C24	0.3 (5)
N1—C1—C2—C3	−1.2 (6)	Ru1—N5—C23—C24	−178.9 (3)
C1—C2—C3—C4	1.3 (6)	C27—N5—C23—C22	179.9 (3)
C1—C2—C3—C6	179.2 (4)	Ru1—N5—C23—C22	0.7 (4)
C2—C3—C4—C5	−0.1 (6)	N4—C22—C23—N5	−2.0 (5)
C6—C3—C4—C5	−178.0 (4)	C21—C22—C23—N5	177.4 (4)
C1—N1—C5—C4	1.5 (5)	N4—C22—C23—C24	177.7 (3)
Ru1—N1—C5—C4	−179.5 (3)	C21—C22—C23—C24	−3.0 (6)
C1—N1—C5—C7	−175.3 (3)	N5—C23—C24—C25	−0.3 (6)
Ru1—N1—C5—C7	3.7 (4)	C22—C23—C24—C25	−179.9 (4)
C3—C4—C5—N1	−1.3 (6)	C23—C24—C25—C26	−0.2 (6)
C3—C4—C5—C7	175.1 (3)	C24—C25—C26—C27	0.6 (6)
C2—C3—C6—O1	−1.8 (6)	C23—N5—C27—C26	0.1 (5)
C4—C3—C6—O1	176.0 (4)	Ru1—N5—C27—C26	179.2 (3)
C2—C3—C6—O2	−179.6 (4)	C25—C26—C27—N5	−0.5 (6)

### Hydrogen-bond geometry (Å, °)

**Table:**

*D*—H···*A*	*D*—H	H···*A*	*D*···*A*	*D*—H···*A*
O2—H2···O1S	0.85	1.75	2.601 (5)	179.
O2—H2···O1T	0.85	1.93	2.545 (18)	129.
O4—H4···O1W^i^	0.85	1.72	2.569 (4)	179.
O1W—H1W···O1^ii^	0.85	1.87	2.720 (4)	179.
O1W—H2W···O2C^iii^	0.85	1.95	2.795 (12)	178.
O1S—H1S···O2A^iv^	0.85	2.14	2.986 (8)	180.
O1T—H1T···O2D^v^	0.85	1.84	2.69 (3)	180.

Symmetry codes: (i) −*x*, −*y*+1, −*z*+1; (ii) −*x*+1, −*y*+2, −*z*+1; (iii) *x*−1, *y*+1, *z*; (iv) −*x*+2, −*y*+1, −*z*+1; (v) −*x*+1, −*y*+1, −*z*+1.
